# Counting motifs in dynamic networks

**DOI:** 10.1186/s12918-018-0533-6

**Published:** 2018-04-11

**Authors:** Kingshuk Mukherjee, Md Mahmudul Hasan, Christina Boucher, Tamer Kahveci

**Affiliations:** Department of Computer and Information Science and Engineering, University of Florida, Gainesville, FL USA

**Keywords:** Biological networks, Dynamic networks, Motif finding

## Abstract

**Background:**

A network motif is a sub-network that occurs frequently in a given network. Detection of such motifs is important since they uncover functions and local properties of the given biological network. Finding motifs is however a computationally challenging task as it requires solving the costly subgraph isomorphism problem. Moreover, the topology of biological networks change over time. These changing networks are called dynamic biological networks. As the network evolves, frequency of each motif in the network also changes. Computing the frequency of a given motif from scratch in a dynamic network as the network topology evolves is infeasible, particularly for large and fast evolving networks.

**Results:**

In this article, we design and develop a scalable method for counting the number of motifs in a dynamic biological network. Our method incrementally updates the frequency of each motif as the underlying network’s topology evolves. Our experiments demonstrate that our method can update the frequency of each motif in orders of magnitude faster than counting the motif embeddings every time the network changes. If the network evolves more frequently, the margin with which our method outperforms the existing static methods, increases.

**Conclusions:**

We evaluated our method extensively using synthetic and real datasets, and show that our method is highly accurate(≥ 96%) and that it can be scaled to large dense networks. The results on real data demonstrate the utility of our method in revealing interesting insights on the evolution of biological processes.

## Background

Biological networks capture complex interactions among molecules (e.g., genes, proteins, etc.) which perform various cellular functions [1]. These networks can be represented as graphs where the set of nodes represents the set of molecules and the set of edges represents the set of known interactions among these molecules. Depending on what the nodes and edges represent (e.g., proteins and signalling events), we classify a biological network as either a protein-protein interaction network, a gene regulatory network, or a signalling network—just to name a few. Interactions among these nodes can be undirected (e.g., protein-protein interaction network) or directed (e.g., gene regulatory network). To understand how cells perform diverse functions, we need to study the networks that model these interactions. Analysing biological networks has proved to be useful in many applications, such as identifying functionally similar pathways in multiple species [2, 3], reconstructing metabolic networks from newly sequenced genome [4] or identifying drug targets [5].

One of the fundamental problems in analyzing biological networks is identification of network motifs. A *network motif* is a small subnetwork that occurs frequently in a given network [6, 7]. These motifs can be viewed as the basic building block of a biological network [6] and thus, uncover functions and local properties of it [8]. Finding network motifs is a computationally hard problem [9]. One way to identify the topological structure of a motif of *n* nodes is to generate all possible subnetwork topologies of *n* nodes and search these topologies in the given target network. This problem becomes intractable as the value of *n* increases since the number of possible topologies grows exponentially with this value. Furthermore, given a motif topology, counting the number of embeddings of this topology is identical to the subgraph isomorphism problem, which is NP-complete [10].

One common formulation to count the number of embeddings of a given motif in a given network is to allow overlap between the subnetworks (i.e. share nodes or edges). Most existing methods for motif counting use this overlap assumption [11–16]. An alternative formulation is to count only disjoint embeddings of each motif—i.e., no two embeddings of the same motif share an edge—in the target network [17]. A third and more restrictive formulation requires that no two embeddings of the same motif share a node in the target network. Counting non overlapping embeddings in a given network requires solving the maximum independent set problem which is NP-complete [9]. The complexities of the motif counting methods also grow rapidly as the number of nodes in the motif and the underlying network increases. Since all these methods try to solve the subgraph isomorphism problem, scaling these methods to large networks remains to be a difficult task.

The motif counting problem, when applied to biological networks, introduces a subtle, yet massive challenge, which is often ignored by most existing studies. This challenge arises due to evolving nature of biological networks. The topology of biological networks change over time. For instance, human embryonic stem cell differentiates into hematopoietic stem cell, then to various other cell types such as liver, kidney, etc. during the development process. Even without cell differentiation, as the chromosomes’ chromatin structures change through folding and unfolding events, different sets of genes get exposed for transcription and thus for interaction. As the network evolves, frequency of each motif in the current network topology can also change. Thus, even if we know the count of a given motif prior to the topological alteration of the network, this number becomes invalid after the alteration. To resolve this issue, we need to update the frequency of each motif such that it effectively mirrors the current snapshot of the network. Methods that compute frequency of motifs assume that the topology of the network remains unchanged once the computation is performed. One trivial way to adopt these methods to dynamically evolving network topologies is to re-compute the frequency of motifs from scratch each time the network evolves. This strategy however renders to be very expensive and impractical particularly for large and highly evolving networks. We need new strategies that quickly adapt to the topological changes as the network evolves.

**Our Contributions.** In this article, we design and develop a scalable method for counting the number of embeddings of given motifs in a dynamic biological network. Briefly, our method first computes the number of embeddings of each motif in the initial network topology. For each motif, we store its embeddings in a list. As the topology of the network evolves through network edit operations (i.e., node (edge) insertion (deletion)), we dynamically process the list of embeddings and update the count of motifs. Lastly, we demonstrate that our method can dynamically update the frequency of each motif in orders of magnitude faster than existing methods, and that it has very high accuracy (e.g., ≥ 96%).

## Preliminaries

### Basic definitions and notation

We restrict our interest to biological networks that can be modeled as a undirected graphs. That said, the methods we develop here can be applied to directed graphs as well. We denote a graph as *G*=(*V*,*E*) with a set of nodes *V* connected by a set of edges *E*. A graph *G*^′^=(*V*^′^,*E*^′^) is a subgraph of *G*=(*V*,*E*) if *V*^′^⊆*V* and *E*^′^ is a subset of edges of *E* connecting the nodes in *V*^′^ (i.e., *E*^′^⊆*E*∩*V*×*V*).

**Fig. 1:**
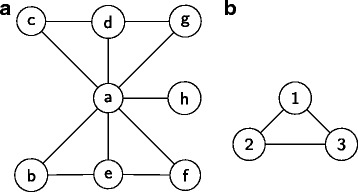
An example to demonstrate pattern and its frequencies

A *path* is a sequence of edges in a graph that constitutes an ordered sequence of distinct nodes. For example, the sequence of nodes [*a*,*b*,*e*,*f*] is a path that is shown in Fig. 1a. We define the *length* of a path as the number of edges on that path. We define the *distance* between two nodes in a graph as the length of the shortest path which connects them.

We next define the concept of connectedness of nodes and graphs. Nodes *u* and *v* of graph *G* are connected if there is a path between *u* and *v* in *G*. Thus, a graph *G*=(*V*,*E*) is connected if all node pairs *u*,*v*∈*V* in *G* are connected. Figure 1a depicts a connected graph. This paper only considers connected subgraphs as motifs. Therefore, in the rest of this article, the term subgraph implies that it is a connected subgraph.

In a given graph *G*=(*V*,*E*), we define the *k-neighborhood* of an edge *e*=(*u*,*v*) as a subgraph *G*^′^=(*V*^′^,*E*^′^), where *E*^′^⊆*E* contains all edges which have at least one incident node whose distance to either *u* or *v* is at most *k*. The set of nodes, *V*^′^⊆*V* contains all nodes connected by *E*^′^.

### Graph isomorphism

We say that two graphs *G*=(*V*_*G*_,*E*_*G*_) and *H*=(*V*_*H*_,*E*_*H*_) are isomorphic if there is a bijection function *π*:*V*_*G*_→*V*_*H*_ such that edge (*u*,*v*)∈*E*_*G*_⇔(*π*(*u*),*π*(*v*))∈*E*_*H*_. Let us denote the graph in Fig. 1a with *G*. Figure 1b presents a graph that forms a triangle. Let us denote this with *P*. There are four subgraphs in *G* which are isomorphic to *P*. These subgraphs consist of the following sets of edges {(*a*,*b*),(*a*,*e*),(*b*,*e*)}, {(*a*,*e*),(*a*,*f*),(*e*,*f*)}, {(*a*,*c*),(*a*,*d*),(*c*,*d*)}, and {(*a*,*d*),(*a*,*g*),(*d*,*g*)}. We denote these subgraphs with *S*_1_, *S*_2_, *S*_3_ and *S*_4_ respectively. We denote the set of all subgraphs in *G* which are isomorphic to *P* with $\mathcal {S} = \{S_{1}, S_{2}, S_{3}, S_{4}\}$

Isomorphism is a transitive relationship. This indicates that all subgraphs in $\mathcal {S}$ are isomorphic to each other. Thus $\mathcal {S}$ defines a topological equivalence class of subgraphs. We represent subgraphs in an equivalence class with a pattern. For example, the triangle pattern *P* represents the equivalence class $\mathcal {S}$. Notice that the nodes of a pattern are unlabeled and it only defines the topology of the subgraph in an equivalence class.

We say that two subgraphs of a given graph *overlap* if they share at least one edge. Otherwise, we call them *disjoint*. For example, in Fig. 1a subgraphs *S*_1_ and *S*_2_ overlap since they share the edge (*a*,*e*). Subgraphs *S*_1_ and *S*_4_ on the other hand are disjoint.

The *frequency* of a pattern *P* in a graph *G* is a function of the set of subgraphs of *G* which are isomorphic to *P*. We call these subgraphs as the *embeddings* of *P* in *G*. The fact that these subgraphs may overlap with each other has led to alternative definitions to the frequency function. The classic definition of frequency ignores overlaps between isomorphic subgraphs. It thus reports the frequency of a pattern as the cardinality of its equivalence class. This frequency function is denoted as the *F*1 count of a pattern [18]. For example, each subgraph in $\mathcal {S}$ is an embedding of the pattern *P* in *G*. Therefore, the *F*1 count of pattern *P* in *G* is four. An alternative measure counts only the disjoint embeddings of a pattern. This frequency is denoted as the *F*2 count. For example, in Fig. 1a the *F*2 count of *P* in *G* is two (e.g., {*S*_1_,*S*3}). A more restrictive version of frequency definition of a pattern does not allow two embeddings to have a common node. This is denoted as the *F*3 count of a pattern. For example, *F*3 count of pattern *P* in *G* is one since node *a* is common to all embeddings in $\mathcal {S}$.

### Dynamic biological networks

Biological networks are inherently dynamic structures. Their topologies change with time through network edit operations. There are four possible network edit operations that perturb the topology of a network, namely (i) node insertion, (ii) node deletion, (iii) edge insertion, and (iv) edge deletion. It is easy to represent node insertion (deletion) using edge insertion (deletion). For example, a node insertion can be represented as a set of edge insertions between existing nodes and the new node. Similarly, a node deletion is equivalent to deleting all edges connected to the deleted node. Formally we define a dynamic network as follows: a sequence of edge deletions and additions $e_{\pi _{1}},e_{\pi _{2}},e_{\pi _{3}}...,e_{\pi _{i}} \forall e_{\pi _{i}} \in V\times V$ are performed on *G*=(*V*,*E*) such that

$G_{i} =\left \{\!\!\! \begin {array}{ll} \,(V, E_{i} = E_{i-1} \cup \{e_{\pi _{i}}\}),& \text {if}~ e_{\pi _{i}}\notin E_{i-1}\\ \,(V, E_{i} = E_{i-1}-\{e_{\pi _{i}}\}), & \text {otherwise} \end {array}\right.$

where *G*_*i*_ represents the topology of the network after the *i*th edge insertion or deletion and *G*_0_=*G*

## Related work

We classify existing methods that are related to our work in two categories: methods for static networks and methods for dynamic networks. The methods on static networks can be broadly divided into two categories: (1) those that count motifs in a single network, and (2) those that count motifs in a set of networks. SUBDUE [19] and GREW [20], for example, find the most frequent subgraphs in a given large network. Complete subgraph finding algorithms are those which are guaranteed to find all subgraphs that satisfy some constraints in a given network. Most subgraph finding algorithms are complete; however, their efficiency falls when they operate on large dense graphs. Algorithms such as SUBDUE and GREW are scalable to an extent but they find only a small number of subgraphs than those discovered by complete algorithms.

The meaning of frequency changes when we consider methods finding frequent subgraphs in a set of networks. If a subgraph exists in a network, the frequency of that subgraph increases by one, irrespective of the number of embedding found in that particular network. FSG [21], gSpan [22] and SPIN [23] fit in this category. FSG generates possible subgraphs by growing them one edge at a time. The number of graphs that contains a certain subgraph is referred to as the support of that subgraph. Frequent subgraphs are reported which exceeds a certain set support threshold. The method becomes expensive as it computes the canonical labels of many redundant subgraphs. gSpan performs a DFS(Depth First Search) and finds out the minimum representation of vertex orderings and then organizes the canonical labels into a hierarchical spanning tree. The frequent subgraphs are discovered by traversing the tree and finding subgraphs which exceed the minimum support. In this way gSpan avoids generating canonical labels for redundant subgraphs. SPIN improves on gSpan by reducing the mining area as it only looks at maximal frequent subgraphs which are not part of larger frequent subgraphs. As mentioned, these methods do not count the number of occurrences of a subgraph in a network but rather check is the subgraph appears at least once in a given network.

CODENSE [24] finds coherent, dense subgraphs in large biological networks. GraphSig [25] mines significant and frequent subgraphs in which graphs are represented as feature vectors. SiS [26] finds subgraphs with the largest probability to appear in a set of biological networks. Most of these methods only focus on the *F*1 count of the patterns (e.g., [11–16]). Others such as MAVISTO [18] computes both *F*2 and *F*3 counts of motifs.

Elhesha et al. [17] finds the *F*2 count of large motifs in a biological network by first computing the *F*2 counts of 4 basic motifs and then joining those basic motifs iteratively to generate larger motifs. Since this paper is one of the most recent papers which computes the *F*2 count of a given motif in a static network, we have used this method for the initial computation of the *F*2 counts and have also compared the running time and accuracy of our method against this method. We explain this in more detail in “Evaluation criteria” section.

There are few methods which focus on dynamic networks and none aim to solve motif counting. Wackersreuther et al. [27] discover frequent subgraphs in dynamic networks. From a time series of graphs they generate a union graph which they call dynamic graph. Each edge in the dynamic graph is denoted with an *edge existence string* which contains the label of that edge at different points of the time series. From the dynamic graph they find subgraphs which share the same common dynamic pattern i.e. they appear and disappear together from the time series of graphs. Qin et al. [28] solve a similar problem but they label the edges 1 or 0 depending on whether that edge exists in a given time point or not. The algorithm finds significant substructures whose edge labels show some fixed properties (patterns of appearance and disappearance from the network) and are within a user defined distance of each other in the dynamic graph.

These methods require the networks at different time points as input and they do all their processing on a union graph constructed from this sequence of networks. Also, these methods only look at the *F*1 counts and do not consider the *F*2 frequency of subgraphs.

## Methods

We begin by describing a method that computes the count of *F*1 and *F*2 for a given motif in a static network. Next, we describe possible network operations that change the topology of the networks, and discuss how to dynamically update the count of *F*1 and *F*2 for each of these operations.

### Motif counting in static networks

Assume that we are given a motif topology denoted with *P*. Given a graph *G*, we want to compute the count of *F*1 and *F*2 of pattern *P* in *G*. Let us denote the set of all embedding of *P* in *G* with *S*. We denote the cardinality of the set *S* (i.e., *F*1 count of *P*) with |*S*|. Recall that the *F*2 count of pattern *P* is the cardinality of the maximal set of embedding where two embeddings do not share edges. We denote such set with $S^{'}\phantom {\dot {i}\!}$. To compute the *F*2 count of *P*, we introduce the concept of an *overlap graph*, which is unique to *P* and *G*. Let us denote the overlap graph with $\phantom {\dot {i}\!}G^{o}=(V^{o}, E^{o})$. Here, each node in *V*^*o*^ corresponds to an embedding of *P* listed in *S*. Let us denote the relationship between the nodes in *V*^*o*^ and the embeddings in *S* with a bijection function *ϕ*:*V*^*o*^→*S*. Each edge (*u*,*v*)∈*E*^*o*^ indicates that the two embeddings *ϕ*(*u*) and *ϕ*(*v*) share at least one edge.

We use the overlap graph to generate the maximal, non-overlapping embedding set $S^{'}\phantom {\dot {i}\!}$ in an iterative fashion. First, we find the node *u*∈*V*^*o*^ with the smallest degree. If there are multiple nodes with the same smallest degree, we randomly select one of them. We insert the corresponding embedding *ϕ*(*u*) into $S^{'}\phantom {\dot {i}\!}$. Since $S^{'}\phantom {\dot {i}\!}$ only contains non-overlapping embeddings, we remove node *u* from *V*^*o*^ along with all the nodes *v*∈*V*^*o*^, such that (*u*,*v*)∈*E*^*o*^. We repeat this process to populate $\phantom {\dot {i}\!}S^{'}$ until *V*^*o*^ becomes the empty set.

### Motif counting in dynamic networks

Let us denote the given network with *G*=(*V*,*E*). Also, let us denote the topology of the network after the *i*th edge insertion or deletion with *G*_*i*_=(*V*,*E*_*i*_). Thus, we have *G*_0_=*G* and ∀*i*≥0,|*E*_*i*_−*E*_*i*−1_|=1. Given a motif topology denoted with pattern *M*, we compute the *F*1 and *F*2 counts of *M* in the initial network *G*_0_ by using the method described in “Motif counting in static networks” subsection. As the network *G* evolves (i.e., new edges are added and/or deleted), the count of *F*1 and *F*2 of *M* can change. Next, we will show an algorithm for efficiently updating the *F*1 and *F*2 counts as the network evolves from *G*_*i*_ to *G*_*i*+1_∀*i*≥0. By repeatedly applying our algorithm, after each network edit operation, the motif count is updated for arbitrarily large sequence of network updates.

#### Updating the *F*1 count

We now describe our method for updating the count of *F*1 of *M* as *G*_*i*_ evolves into *G*_*i*+1_. We assume that *F*1 for *G*_*i*_ is known. Our algorithm for updating *F*1 relies on initially constructing and maintaining an auxiliary data structure that allows for the embeddings containing an edge to be efficiently queried. Thus, at the beginning of our algorithm, we find all embeddings of a given motif *M* in the initial network *G*_0_. After finding these embeddings, we create a list of embeddings for each edge *e*∈*E*, denoted as *D*_*e*_, which stores all embeddings that contain *e*. That is, for a motif *M*, let *m* be an embedding in a given network. Then *m*∈*D*_*e*_ if *e*∈*m*. This data structure, which we refer to as the *edge compressed bitmap*, is updated each time an edge is either added or deleted. The *F*1 is then updated based on the *edge compressed bitmap*.

Suppose that as the network *G*_*i*_ evolves to *G*_*i*+1_ the *e*∈*E*_*i*_ is deleted. This reduces the *F*1 count of motif *M*, if the deleted edge is a part of embeddings of *M*. From the *edge compressed bitmap*, we find the set of embeddings of *M* which contain *e*. We remove this set (*D*_*e*_) from the edge compressed bitmap and reduce the *F*1 count of *M* by the cardinality of this set.

Next, assume that an edge *e*∉*E*_*i*_ is added to *G*_*i*_. Unlike the edge deletion, prior to this update, we do not know whether *e* is a part of an embedding of *M* in *G*_*i*+1_. We locate such embeddings of *M* in *G*_*i*+1_ as follows. Let us denote the diameter of *M* with *k*. We search the *k-neighborhood* of *e* in *G*_*i*+1_. The set of embeddings of *M* which contains *e* can be formed with its neighboring edges. We add this set to the edge compressed bitmap and increase the *F*1 count of *M* by the cardinality of the set of new embeddings.

#### Updating the *F*2 count

After updating the *F*1 count, we proceed to update the *F*2 count. Updating the *F*2 count is more challenging than updating *F*1 because computing the count of *F*2 is NP-complete [9] and the methods used are heuristics. As a result, the *F*2 count we compute even for a single static network may deviate from the optimal result. We would like to minimize the additional errors introduced by dynamic updates.

First, we assume that we have already computed the *F*1 and *F*2 counts of the given motif *M* in *G*_*i*_ and the *F*1 count of *M* in *G*_*i*+1_. Next, we describe how we update the *F*2 count for *G*_*i*+1_. There are following two possible scenarios: (1) an edge has been deleted from *G*_*i*_, and (2) and edge has been added to *G*_*i*_. In the first scenario, the removal of an edge *e* from *G*_*i*_ will cause the *F*2 count to either remain the same or decrease by one. The former case occurs when none of the embeddings in the set *D*_*e*_ contribute to the *F*2 count in *G*_*i*_. The latter case occurs when one of the embeddings from the set *D*_*e*_, contributes to the *F*2 count of *G*_*i*_. Let us denote that embedding with *X* (*X*∈*D*_*e*_). After removing *e*, the embedding *X* does not exist in *G*_*i*+1_. This reduces the *F*2 count of *M* by one. However, it is possible that there is another embedding (say *Y*), which can be included in the *F*2 count for *G*_*i*+1_ to replace *X*. For this to happen, *Y* must satisfy two conditions: (i) *Y* overlaps with *X*, and (ii) *Y* does not overlap with any other embedding included in the *F*2 count of *M* in *G*_*i*_. If such an embedding *Y* exists, we include it in the *F*2 set. Thus the *F*2 count remains unaltered. Otherwise the *F*2 count decreases by one.

In order to identify any embedding *Y* that satisfies the two condition above, we explore the *neighbors* of *X* in the overlap graph. Recall that the *neighbors* of an embedding in the overlap graph are those embeddings of *M* which share at least one common edge with that embedding. If say, *X* consists of edges *e*_1_, *e*_2_ and *e*_3_ then the *neighbors* of *X* will be the union of sets $D_{e_{1}}$, $D_{e_{2}}$ and $D_{e_{3}}$.

From the set of *neighbors* of *X*, we consider each embedding and check if they can be included in the updated *F*2 count. If an embedding *Y* in that set, has all of its edges free then we include it in the *F*2 set for *G*_*i*+1_. Therefore, if such an embedding *Y* exists, the *F*2 count remains unaltered as the inclusion of *Y* compensates for the deletion of *X*. Otherwise we decrease the *F*2 count by one.

Assume that an edge *e*, where *E*_*i*+1_−*E*_*i*_={*e*} is added *G*_*i*_.This addition will either increase the *F*2 count of *M* in *G*_*i*+1_ by one or has no influence. The new edge can form new embeddings of *M* in *G*_*i*+1_. We explain how we obtain such new embeddings in “Updating the *F*1 count” section. We then check if any of these new embeddings can be included in the updated *F*2 count. To do this, we consider each new embedding, and check if all of its edges are uninvolved in the *F*2 count (they could be involved in the *F*2 count with other embeddings). If such an embedding exists, we include it in the *F*2 set and increase the *F*2 count by one.

## Results

### Datasets

We use synthetic and real datasets to evaluate our method. First, we describe our synthetic dataset. We use synthetic networks to observe the performance of our method by varying a broad suite of parameters, namely the number of nodes, the average number of edges per node in the network and the topology model. We call the number of nodes in the networks and average number of edges per node as network *size* and *degree* respectively. We generate undirected random networks using three generative models, namely (i) Erdös-Rényi (ER) [29], ii) Watts-Strogatz (WS) [30] and (iii) Barabási-Albert (BA) [31]. We experiment with networks of different sizes and degree values. We generate networks with sizes ranging from 1000 to 5000 at increments of 1000. For each network size, we set the network degree to ten, fifteen and twenty. For each parameter combination, we randomly generate 10 undirected synthetic networks for each model. In total, we generated 450 (i.e., 3 × 5 × 3 × 10) synthetic networks.

**Fig. 2:**
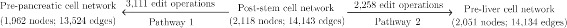
Summary of simulated networks from real data

Next, we describe our secondary dataset. We generate gene co-expression network using the transcription data from Rivera-Mulia et al. [32]. This dataset contains the gene expressions for 19 human cell lines obtained at various stages of development from embriyonic stem cell to various cells. Table 1 lists these cell lines. From these cell lines, we construct co-expression networks at three different stages of development (i) *Post stem cell* uses all the cell lines except the stem cells. (ii) *Pre-pancreatic cell* includes all cell lines but the pancreas cells. (iii) *Pre-liver cell* contains all cell lines except liver cells. We use the datasets (i) and (ii) above to build a pathway of two networks for pancreas cell development. We use (i) and (iii) to build a second pathway for liver development. For the first pathway above we construct two networks as follows. We compute the Pearson’s correlation between the transcriptions of all gene pairs in the two datasets ((i) and (ii)). We use two correlation thresholds; *stringent cutoff* set to 0.95, and a *relaxed cutoff* set to 0.75. Assume that the post stem cell dataset is the primary and the Pre-pancreatic cell dataset is the secondary dataset. Given a pair of genes *u* and *v* we include an edge between them in the primary dataset network if one of the two conditions hold: (1) correlation between *u* and *v* within the primary dataset is above the stringent cutoff, or (2) correlation between *u* and *v* within the secondary dataset is above the stringent cutoff while that within the primary dataset is above the relaxed cutoff. We then construct the network for the Pre-pancreatic dataset similarly by assuming it to be the primary and the post stem cell dataset as the secondary one. We repeat the same process to construct another sequence of two networks for the liver pathway. The details of the three simulated networks and two pathways are summarized in Fig. 2.

**Table 1:** Cell lines used in our experiments

Cell line	Cell type
ESC_Cyt49	ESC
ESC_H9	ESC
NC_Cyt49	Neural crest
NC_H9	Neural crest
MSC_H9	MSCs
NPC_H9	NPCs
LPM_H9	Lateral plate mesodem
Splanc_Cyt49	Splanchnic mesoderm
Splanc_H9	Splanchnic mesoderm
Epic_Cyt49	Mesothelium
Epic_H9	Mesothelium
SM_H9	Smooth muscle
DE_Cyt49	Definitive endoderm
Liver_d5_Cyt49	Immature hepatic
Liver_d8_Cyt49	Hepatoblast
Liver_d16_Cyt49	Liver
Panc_d5_Cyt49	Primitive gut
Panc_d8_Cyt49	Posterior foregut
Panc_d12_Cyt49	Pancreas

### Evaluation criteria

We simulate dynamic changes in the synthetic networks as follows. For each network, we perform a sequence of 1000 edit operations (i.e., edge insertion and deletion). We conduct the topological perturbations using the degree preserving edge shuffling method [33]. Each shuffle takes two edges (*a*,*b*) and (*u*,*v*), such that they share no nodes. It then replaces these edges with (*a*,*v*) and (*u*,*b*). Thus, one edge shuffling introduces two edge insertions and two edge deletions in a random order. Such edge shuffling ensures that all the nodes in the network preserves their degrees.

**Fig. 3:**
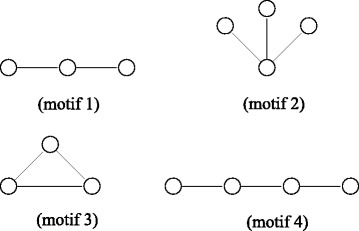
Motifs used in the experiment

In our experiments, we use a small set of motifs that have been used frequently in literature. Figure 3 shows these motifs. Note that these are all possible topologies with two and three edges. We call these topologies the *basic* motifs. Having said that, our method is generic and can be employed to count any motif topology. Given a sequence of network edit operations, we first compute the number of embeddings for each motif in Fig. 3 before applying these edit operations. We do this using the method presented in Elhesha et al. [17] as it is one of the most recent papers which compute the *F*2 frequency of motifs in a static network. We then apply each edit operation in the order it was given to update the network topology. After each edit operation, we dynamically update the count of each motif using our method.

We compare our method against Elhesha et al. [17] as it also computes the *F*2 count of the four basic motifs. We evaluate our method in terms of its accuracy and running time. All experiments were performed on a server with 3 GB RAM and AMD Opteron dual core processors (2.2 GHz) running Linux. Notice that unlike our method, Elhesha et al. computes the *F*2 count from scratch when the network topology changes. Thus, at any point in the evolution, we consider their count as the ground truth. We report the accuracy of our method as the ratio of the *F*2 count of our method to the *F*2 count returned by their method at that instance of the network. We measure the running time of the static method for the initial network topology and denote it with *static time*. We also measure the running time of our method after each edit operation and denote it with *dynamic time*.

### Running time on the synthetic dataset

#### Effect of network model

**Fig. 4:**
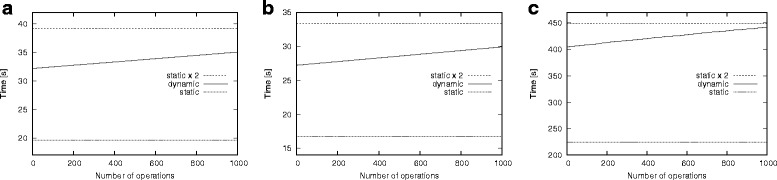
Effect of network models on the running time. The three models are (i) Erdös-Rényi (ER), ii) Watts-Strogatz (WS) and (iii) Barabási-Albert (BA). The lower dotted line represents the running time for the static algorithm and the upper dotted line represents the twice the running time for the static algorithm. The solid line shows the running time for our dynamic algorithm as it computes the counts of the initial network and then updates them as the network evolves from *G*_0_ to *G*_1000_. **a** ER, **b** WS, **c** BA

We first investigate the effects of different network models on the running time of our algorithm. We set the network size to 5000 nodes and the average node degree to 15. We perform 1000 random edit operations and dynamically update the motif counts for all motif topologies in Fig. 3. Figure 4 presents the results.

We observe that the running time of our method for all three models are significantly less than that of the static method. The static method needs to recompute the *F*2 count from scratch every time the network topology changes. To illustrate this, we plot the cost of running the static algorithm twice, i.e., one for initial topology and the other for an arbitrary intermediate topology as the network evolves. We observe that, despite the additional cost our method incurs for initialization, the total cost of our method for 1000 dynamic topologies is less than that of the static method for only two topologies. More specifically our method updates the motif count for 1000 evolving topologies more than four times faster than the static method on a single network topology.

We observe that both static and dynamic methods incur the largest running time for the BA model. This is because the degree distribution in the BA model forces motif counting algorithms to evaluate many possibilities not encountered in other models, hence the increase in the running time. We observe much faster running time for the other two models.

#### Effect of network Size

**Fig. 5:**
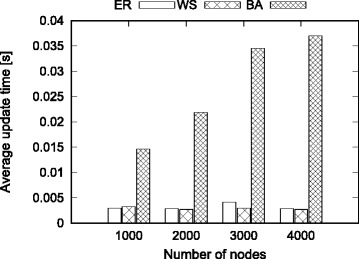
Effect of network size on average update time for different random network models

In this experiment, we fix the average degree of the networks to 15, and vary the number of nodes from 1000 to 4000 with increment of 1000. We perform the experiments for each motif and random network model combination. We process 1000 random edit operations and dynamically update the motif counts. We compute the total update time as the processing time starting from the first edit operation until the end of the last edit operation. We compute the average update time as the total update time divided by the number of edit operations. Figure 5 shows the results.

We observe that the average update time of our method is very small even for the largest network size. This shows the effectiveness of using dynamic update in the motif counting problem.

#### Effect of average degree

**Fig. 6:**
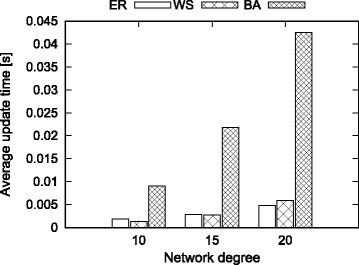
Effect of node degree on average update time

In this experiment, we fix the network size to 4000 nodes and set the average node degree to 10, 15 and 20 respectively. We perform experiments for each motif and random network model. We process 1000 random edit operations and dynamically update the motif counts. We report the average update time for the edit operations. Figure 6 presents the results.

We observe that the average update time of our method is very small for all degree values. Average update time increases as the node degrees increase. Among the three models, we observe that the BA model takes longer time to update and the other two models show similar performance.

These experiments support our conjecture that dynamic update time per edit operation is very small for all parameter combinations. It scales well to networks with large sizes and node degrees. The potency of our method is important in evolving biological networks where running static method after each topological change is not practical.

### Accuracy on the synthetic dataset

**Fig. 7:**
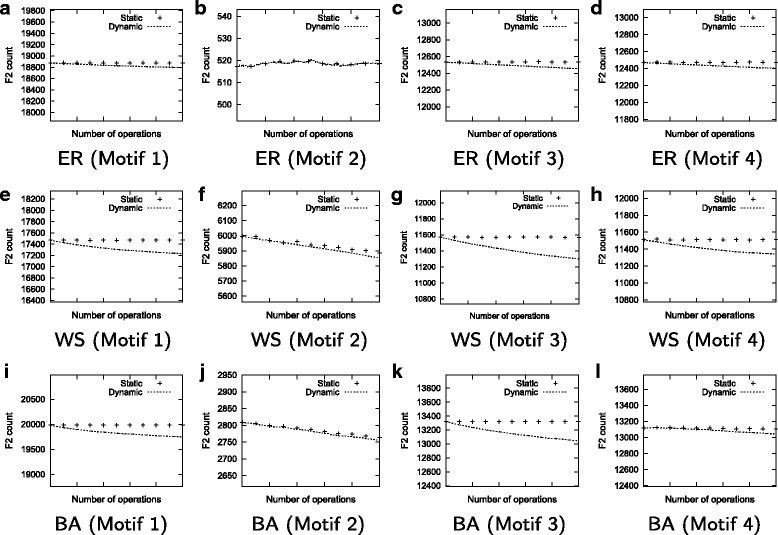
Number of embeddings found by our dynamic method and static method as the network evolves through 1000 edit operations. **a** ER (Motif 1), **b** ER (Motif 2), **c** ER (Motif 3), **d** ER (Motif 4), **e** WS (Motif 1), **f** WS (Motif 2), **g** WS (Motif 3), **h** WS (Motif 4), **i** BA (Motif 1), **j** BA (Motif 2), **k** BA (Motif 3), **l** BA (Motif 4)

In the previous section, we show that our method dynamically updates the motif count very fast in all parameter combinations. Recall however that it is possible that our dynamic method may yield fewer *F*2 count as compared to the static method. Faster running time is desirable only if our method produces accurate results. Here, we evaluate the accuracy of our method as compared to the static one. We run our experiments with network size and degree set to 5000 and 15 respectively. To compare with static method, we also run static method [17] after every 100 edit operations. Figure 7 presents the results.

We observe that our method is highly accurate for all motifs and random network models. The *F*2 count reported by our method is very close to that of the static method. As the number of edit operation increases, the gap between the two *F*2 counts gradually grow. This is expected since the static method computes the number of embeddings from scratch, whereas our method dynamically updates the count from the initial computation. That said, the gap remains to be negligible even after applying 1000 edit operations.

#### Effect of network size

**Fig. 8:**
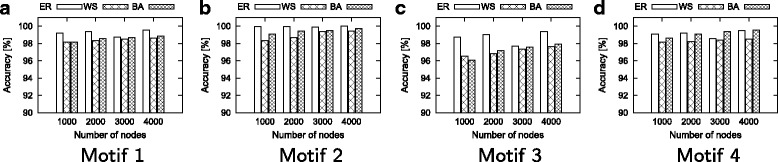
Accuracy found by our dynamic method and static method. **a** Motif 1, **b** Motif 2, **c** Motif 3, **d** Motif 4

In this experiment, we fix the average degree of the networks to 15, and vary the network sizes from 2000 to 5000 with increment of 1000. In this experiment, we dynamically compute the motif counts after each of the 1000 edit operations. In the final topology, we also compute the results using the static method. We report the accuracy of our method for each motif and random model combinations. Figure 8 shows the results.

Our results demonstrate that our method is highly accurate for all network sizes, motifs, and random network models. The accuracy of our method is over 95% in all of our experiments. This shows the robustness of our method.

#### Effect of average degree

**Fig. 9:**
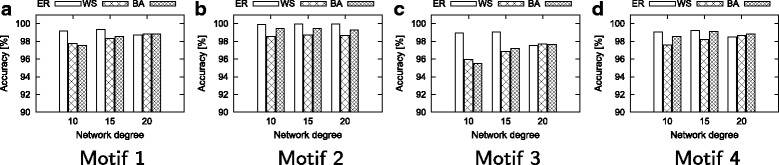
Accuracy found by our dynamic method and static method. **a** Motif 1, **b** Motif 2, **c** Motif 3, **d** Motif 4

In this experiment, we fix the network size to 5000 and vary the degrees to 10, 15 and 20. We dynamically compute the motif counts after each of the 1000 edit operations. In the final topology, we also compute the results using the static method. Here, we report the accuracy of our method in different random models. Figure 9 shows the results.

Our method is highly accurate for all degree values. For instance, accuracy values of motifs one, two and four are over 97% for each network size. For all degree and random network combinations, our method counts the motifs with more than 95% accuracy.

In summary, our method yields highly accurate results for all network sizes, density values, motif topologies, and random network models. These results suggest that our method is robust to a broad spectrum of parameter settings.

### Evaluation on the real dataset

We perform experiments on two pathways using the gene co-expression dataset described earlier in “Datasets” section. The first one is from post stem cell network to Pre pancreatic cell network. The second one is from post-stem cell network to pre-liver cell network. We consider the first network in each pathway as the *starting network* and the second one as the *final network* in the dynamic evolution. The difference in the set of edges between the starting and the final networks is the set of edit operations that the starting network goes through to evolve into the final network. As the order in which these edit operations take place are not known, we rank them randomly. We then use the static algorithm on the starting network to find the motif embeddings for the same motifs used in “Evaluation criteria” section. We then apply the edit operations in the given order and update the motif counts and embeddings. We also find all the motif embeddings and the F2 count of the motif in the final network using the static algorithm for comparison.

#### Evaluation of running time

**Fig. 10:**
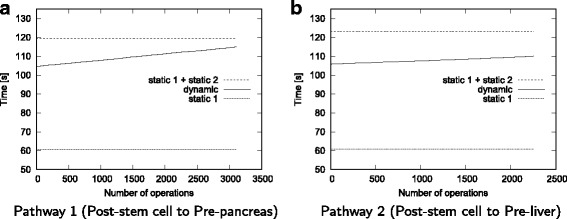
Time analysis for real experiments. The lower dotted line represents the time taken by the Static algorithm to compute the counts in the *starting network* in the pathway. The upper dotted line represents the time taken by the static algorithm to compute the counts in the starting network plus the time taken by the static algorithm to compute the counts in the *final network*. The solid line represents the time taken by our dynamic algorithm to update the counts from the *starting network* to the *final network* as it goes through the network edit operations (plotted in X-axis). **a** Pathway 1 (Post-stem cell to Pre-pancreas), **b** Pathway 2 (Post-stem cell to Pre-liver)

Figure 10 presents the running time results for our method on the two pathways. For comparison, it also shows the running time of the static algorithm when it is applied to only the starting network, and both starting and final networks once. Our results demonstrate that our dynamic algorithm takes less time to update the *F*2 counts for over thousands of edit operations as compared to running the static algorithm once on two network instances only (the starting and final networks). This is a dramatic improvement as our method computes the motif count for not only those two networks but also thousands of network topologies in between these two states of networks. In other words our algorithm can identify motif counts for thousands of networks while the static algorithm fails to do that without running it from scratch. We observe that while the static algorithm takes around 60 s per network to count motifs, our dynamic algorithm updates the motif count in only a few milliseconds per intermediate network.

#### Evaluation of accuracy

**Fig. 11:**
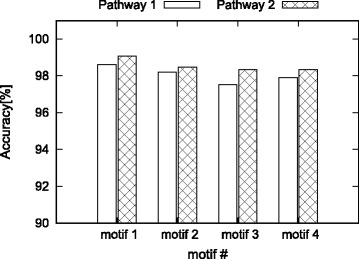
Accuracy of the dynamic algorithm for the real networks

The performance improvement is desirable only if the algorithm remains accurate. Here, we evaluate whether our method correctly computes motif counts. We focus on the final network of each pathway for this purpose. We run our algorithm to dynamically update the *F*2 motif count until we reach to the final network. Let us denote this count with *c*_dynamic_. We then compute the same count using the static algorithm when it is applied on the final network only. Let us denote this count with *c*_static_. We report the accuracy as the percentage of the motif count our method finds over the static one (i,e., accuracy = 100×*c*_dynamic_/*c*_static_. Figure 11 presents the results.

Similar to the synthetic datasets, we observe that our dynamic algorithm is highly accurate on the real datasets as well. For all the motifs tested, the accuracy is over 97% for both pathways. We observe higher accuracy for the second pathway. This is possibly because the number of network edit operations for the second patway is lesser than that of the first one (see Fig. 10). The results are very promising for our method as both pathways are outcomes of a large number of edit operations.

## Discussion

From the results on the simulated dataset we observe that the running time of our method for all three models are dramatically less than that of the static method. For the initial network topology, the dynamic method spends a little more time than the static method to compute the *F*2 count. This is because it initializes the relevant data structures which are needed to quickly update the *F*2 count as the network evolves. Once the data structures are set up, however, the cost of dynamic update becomes negligible. This demonstrates the key advantage of our dynamic method over the static one. The average update time grows very slowly as the network size increases. This suggests that our method scales to large networks and very large number of network edit operations. Among the three models, we observe that relative to the other two models, the BA model takes longer time to update. This is because the networks in BA model yields significantly more motif instances. The potency of our method is important in evolving biological networks where running static method after each topological change is not practical.

We also observe that our method is highly accurate for all motifs and random network models. The accuracy of our method remains stable with increasing network size while the accuracy values for BA model increase as the network size grows. Since the BA model represents the biological networks well, these results promise the applicability of our method in real networks. We also observe that our method is stable for growing node degree values.

Our experiments with the real dataset shows that our methods have similar efficacy on real data as well. Therefore, it can be used on biological dynamic networks to uncover interesting observations on the evolution of biological processes.

## Conclusions

Several approaches exist to compute the number of embeddings for a given motif in static network; however, no such method exists for dynamic networks. In this article, we address this problem and describe a method that incrementally updates the motif count as the network changes its topology. We evaluated our method extensively using synthetic and real datasets, and show that our method is highly accurate and that it can be scaled to large dense networks. The results on real data demonstrate the utility of our method in revealing interesting insights on the evolution of biological processes.
